# Use of Wearable Inertial Sensors to Assess Trunk and Cervical Postures Among Surgeons: Effect of Surgical Specialties and Roles

**DOI:** 10.3390/bioengineering12030299

**Published:** 2025-03-15

**Authors:** Giulia Casu, Micaela Porta, Luigi Isaia Lecca, Alessandro Murru, Fabio Medas, Massimiliano Pau, Marcello Campagna

**Affiliations:** 1 Department of Mechanical, Chemical and Materials Engineering, University of Cagliari, Piazza d’Armi, 09123 Cagliari, Italy; giulia.casu96@unica.it (G.C.); micaela.porta@unica.it (M.P.); 2 Department of Medical Sciences and Public Health, University of Cagliari, 09123 Cagliari, Italy; luigi.lecca@unica.it (L.I.L.); alessandro.murru2@unica.it (A.M.); mcampagna@unica.it (M.C.); 3 Department of Surgical Sciences, University of Cagliari, 09123 Cagliari, Italy; fabiomedas@unica.it

**Keywords:** wearable sensors, surgery, non-neutral postures, ergonomic risk, in-field measurement

## Abstract

This study aimed to quantitatively assess trunk and cervical non-neutral postures assumed by surgeons during the performance of routine open procedures. Indeed, musculoskeletal disorders are frequently reported by surgeons, especially at the head and neck level, due to the prolonged time spent in ergonomically challenging postures. Therefore, the posture of fourteen surgeons was monitored using wearable inertial sensors (and processed according to the ISO 11226 standard) by considering the effect of different surgical specialties (thyroid vs. breast) and roles (primary vs. assistants). Overall, surgeons spent most of their time in a standing posture, remaining within the acceptable limits of trunk flexion. More concerning results were observed analyzing the time spent in static head flexion and lateral bending (~72% and 48% of the time, respectively). Assistants, compared with primary surgeons, spent more than twice as much time in extreme neck flexion, although this was only when performing thyroid surgeries. The opposite was observed during breast surgeries. By spending most of their time in a standing posture with extreme forward neck flexion, surgeons are exposed to a high ergonomic risk, especially when frequently performing thyroid surgeries. The assumed role appeared to influence postural loading, with an effect that varies according to the surgical specialty.

## 1. Introduction

Among the wide range of healthcare professionals, surgeons have been recognized as one of the roles most exposed to the risk of developing work-related musculoskeletal disorders (WMSDs). Indeed, they frequently operate under time pressure on anatomically challenging and relatively inaccessible body areas. In the long term, such conditions pose significant physical and mental strain [1], which are likely to affect their capacity to provide optimal care. Once present, WMSDs induce disturbance in sleep and mental concentration, thus leading to an overall decrease in the quality of life [2]. Ultimately, this problem potentially has a huge impact on patients’ health and society, as it may lead to staff shortages, thus creating longer waiting lists and increased healthcare costs [3].

Regardless of surgery specialties, surgeons are often required to apply forceful exertions to make the surgical area of interest optimally exposed and accessible [1]. For this purpose, surgeons adopt ergonomically challenging postures characterized by sustained shoulders abductions [4] and poor cervical angle [5], thus exposing arm and back muscles to long periods of activity that are likely to induce neuromuscular fatigue, as reported by previous quantitative studies carried out using wearable sensors [6]. Given the relevant and continuous working load (approximately 8–10 h per day and over 50 per week [2]), cumulative exposure to sustained isometric muscle contraction and non-neutral postures typically leads to injuries involving various body districts, with the prevalence of WMSDs ranging from 23% to 94%, depending on the type of surgery performed [7]. Aaron and colleagues [5], in their recent study involving 389 surgeons across different subspecialties, reported that 69.7% suffered from musculoskeletal pain due to the self-reported excessive time spent in a standing posture, with the most affected regions including the neck (82.9%), lower back (68.1%), shoulder (57.8%), and upper back (52.6%) [1,5,8]. Despite the phenomenon being, overall, under-reported, the growing interest of the scientific community as well as the geographical distribution of the researchers who have carried out studies on WMSDs among surgeons suggest that this problem is widespread worldwide, with peaks in the USA [9], which is probably due to its insurance-based healthcare management and large population.

Although such issues are shared among all surgical specialties, the risk of experiencing WMSDs varies according to the type of surgery performed [5]. In particular, it has been recognized that endocrine surgeons report the highest incidence of musculoskeletal symptoms, with pain and stiffness being the most prevalent [10]. Thyroid surgery, which is the most common endocrine surgery [10], is usually performed by otolaryngologists, who reported musculoskeletal symptoms with a prevalence of approximately 60% among those who routinely performed open head and neck procedures, such as thyroidectomy or parotidectomy [11]. For this reason, otolaryngologists are classified among the subspecialties with the highest rates of WMSDs [10]. The higher ergonomic risk to which endocrine surgeons are exposed is likely related to operating in particularly confined spaces, which may increase the propensity for poor posture and the awkward positioning/handling of instruments [10]. On the contrary, surgeons involved in general open surgery (including procedures in the abdominal cavity, plastic, gynecology and breast surgery), by focusing in a typically more easily accessible surgical area, reported proportionately lower intraoperative discomfort compared to otolaryngologists, especially at the cervical and lumbar regions [5].

The gradual replacement of open surgery with minimally invasive surgeries (MISs, e.g., laparoscopic, robotic, and endoscopic surgeries), the use of ergonomic tools such as operating microscopes [12] and the adoption of surgical robots [13] that has been seen in recent decades was expected to minimize the need to maintain extreme non-neutral postures (especially of the neck) that are common during the positioning and/or handling of surgical instruments. Nevertheless, the required use of supplemental equipment (e.g., loupes) and the need to establish a proper surgical field visualization paradoxically made surgeons performing MISs at even higher risk to experience WMSDs, with respect to those performing open surgery [8].

While it is well-established that surgeons from various subspecialties face different risks of developing WMSDs, the impact of the specific role assumed during surgery remains unclear. In fact, most of the currently available studies were focused on primary surgical tasks, thus neglecting the work of surgical assistants, who are often considered to perform passive tasks [14]. However, as they are required to hold instruments like retractors, suction devices, etc., to support the work of the primary surgeon in accessing and operating in the surgical area, assistants may experience an equally important mechanical strain. In this regard, Yurteri-Kaplan [15] observed that, during gynecological surgeries, surgical assistants were more exposed to sustained awkward trunk, neck and shoulder postures than primary surgeons, even if they had a lower frequency of movement. Moreover, postural instability and high levels of muscle activation were observed during the performance of laparoscopic assistant tasks, such as camera driving and equipment retraction, but not during primary operative tasks [14,16]. Therefore, to date, it remains unclear whether the role assumed by the surgeon throughout the operation can represent a relevant factor in the risk of developing WMSDs.

Considering that WMSDs can substantially influence surgeons’ work performance, causing practice restriction or modification, absenteeism, and in the worst-case scenario, early retirement [17], the present study aims to provide quantitative data to support ergonomic assessment for MSD prevention. In this study, we used wearable inertial sensors to assess the exposure to trunk and cervical non-neutral posture in surgeons during the performance of actual surgical procedures. In particular, we analyzed different surgeries (i.e., thyroid vs. breast surgery) and roles (primary surgeons vs. surgical assistants) to investigate their influence in the exposure to non-neutral posture. Moreover, in order to provide elements that are useful for a practical ergonomic assessment, the instrumental data were interpreted according to the requirement of the International Standard ISO 11226 for the assessment of exposure to static non-neutral postures [18].

## 2. Materials and Methods

### 2.1. Participants

For the purpose of the present study, 14 surgical procedures were monitored for their entire duration at the Department of Surgery of the “Duilio Casula” University Hospital (AOU) of Cagliari (Italy). Half of them were breast surgeries (including unilateral mastectomy with or without sentinel lymph node dissection) and the remaining half were thyroid surgeries (i.e., total thyroidectomy procedures). The study involved two surgeons per procedure, respectively, assigned to the role of the primary surgeon and surgical assistant, whose demographic and anthropometric characteristics are shown in Table 1. The choice of their role was left to the surgeon’s discretion, based on their experience and expertise in the operating room. Specifically, the primary surgeon led the procedure, performed key surgical steps and ensured patient safety, while the surgical assistant supported the primary surgeon by providing instruments and performing tasks like tissue retraction and suturing. A properly trained team of occupational physicians visually monitored the surgeries by noting in real-time any movement and/or change in position, with respect to the operating table. Their role was also essential in reporting (and, when possible, preventing) any change in sensor position which could possibly affect data validity. Before data collection, participants received a detailed explanation of the purposes and methodology of the study and then read and signed an informed consent form. The study was carried out in compliance with the ethical principles for research involving human subjects expressed in the Declaration of Helsinki and its later amendments and received approval from the Ethical Commission of the University of Cagliari (UniCa-Prot. n. 0112541, 1 June 2023).

### 2.2. Data Collection and Processing

**Figure 1:**
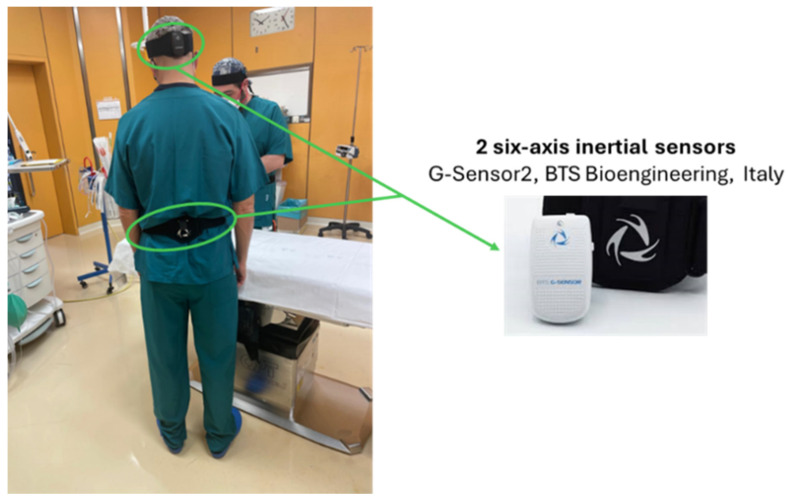
Example of sensor placement. For details, please refer to the main text.

The trunk, head and neck posture of each surgeon was assessed by means of a simplified measurement set-up (shown in Figure 1) characterized by two commercially available 6-axis inertial measurement units (IMUs, 3-axes accelerometer combined with a 3-axes gyroscope, G-Sensor2, BTS Bioengineering, Milan, Italy), previously employed in similar studies [19,20]. One IMU was placed, using an anthropometrically adjustable semi-elastic belt, approximately in correspondence with the first lumbar vertebrae [21] to quantify trunk posture (i.e., trunk flexion and lateral bending). The second IMU, placed at the back of the head [22], was used to monitor head posture. Both IMUs were set to collect data onboard (at 50 Hz frequency) for the entire duration of the procedures, which typically was in the range 0.4–2.0 h.

**Figure 2:**
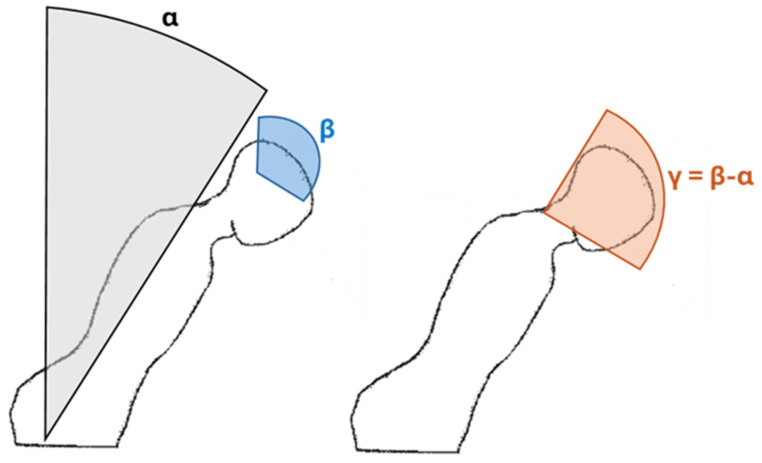
Representation of trunk (α, in gray) and head (β, in blue) flexion calculated from the two IMUs; neck flexion is indicated in orange and calculated as the difference between trunk and head flexion (γ = β − α). Adapted from ISO 11226.

At the beginning of the experimental session, the surgeons were asked to stand still for 10 s in a neutral, upright posture in order to capture subject-specific angular offset and remove it from the acquired data to obtain absolute angles for each monitored body district. Specifically, raw data from each IMU, representing Cardan angles (i.e., roll, pitch and yaw), were processed according to the standard ISO 11226 using dedicated software developed in Matlab (R2020b, MathWorks, Natick, MA, USA) to obtain trunk flexion–extension (FE) and lateral bending (LB) (from the first IMU), head FE and LB (from the second IMU) and neck FE. In particular, neck FE was quantified as the difference between the orientation of the trunk and head IMUs that was synchronized at the beginning of the data collection (Figure 2). Finally, to obtain easily interpretable results, angular data were further processed to calculate the percentage of time spent in a static (e.g., longer than 4 s) non-neutral posture of the three analyzed body districts with respect to the entire surgery duration, according to the classes of risk indicated in the ISO 11226 (Table 2).

### 2.3. Statistical Analysis

Descriptive statistics for each considered parameter (e.g., percentage of time spent in non-recommended static trunk FE and LB, head FE and LB, neck FE) were calculated in terms of mean and standard deviation (SD). The possible existing differences associated with the surgery type (i.e., breast vs. thyroid) and the role (i.e., primary surgeon vs. surgical assistant) in terms of biomechanical exposure were explored using two independent multivariate analyses of variance (MANOVA), as follows:

- Two-way MANOVA carried out by setting the surgery (i.e., breast vs. thyroid) and the role (i.e., primary surgeon vs. surgical assistant) as independent variables and trunk posture parameters (i.e., percentage of time spent in different classes of trunk FE and LB) as dependent variables;
- Two-way MANOVA carried out by setting the surgery (i.e., breast vs. thyroid) and the role (i.e., primary surgeon vs. surgical assistant) as independent variables and head/neck posture parameters (i.e., percentage of time spent in different classes of head FE, head LB, and neck FE) as dependent variables.

Two-way ANOVAs were then performed as a post hoc analysis by reducing the level of significance after a Bonferroni correction for multiple comparisons. All statistical analyses were performed using SPSS software (v.20, IBM, Armonk, NY, USA) by setting the level of significance at *p* = 0.05 and using the eta-squared (η^2^) coefficient to assess the size effect.

## 3. Results

The following paragraphs summarize the results of the biomechanical exposure of the monitored surgeons, classified according to the type of surgery performed (breast and thyroid) and the assumed role (primary surgeon and surgical assistant). In addition, in the Supplementary Material, Table S1 (and graphically Figure S1) reported the percentage of time spent in non-neutral posture of the trunk, head and neck according to the classes of risk proposed by ISO 111226, for each specialty and role.

Occasionally, primary surgeons left the operating room after completing the critical phases of the procedure. For this reason, assistants were monitored for a longer duration (1.17 and 1.72 h, respectively, for breast and thyroid surgeries) compared to primary surgeons (1.11 and 1.29, respectively, for breast and thyroid surgeries).

### 3.1. Trunk

**Figure 3:**
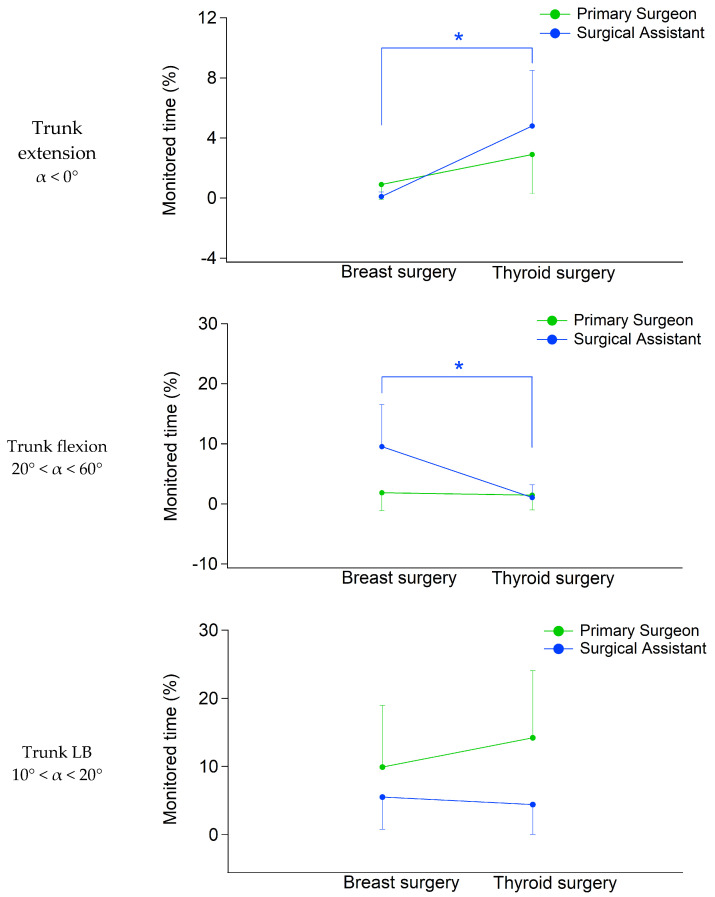
Average trunk FE and LB among different surgeries and roles, following the classes of risk proposed by ISO 11226. Errors bars indicate SDs and the symbol * indicates a significant difference between surgery groups within the same role.

Overall, surgeons spent most of the procedure time in static (e.g., longer than 4 s) trunk posture, with an average flexion angle of 26° and LB of 6°, rarely (only 15/28 surgeons for a maximum duration of 4 min) performing trunk movement exceeding the not-recommended trunk posture (i.e., flexions > 60° and LB > 20°). The statistical analysis detected a significant main effect of the surgery type [F(6,18) = 3.423, *p* = 0.020, Wilks’λ = 0.467, η^2^ = 0.533]. In particular, breast surgeons spent on average 5.4% of the monitored time in static trunk flexion above 20° (0.8% in non-static condition). In parallel, thyroid surgeons spent on average 1.3% of the monitored time in static trunk flexion above 20° (0.5% in non-static condition). A significant difference was also found for the average time spent in static trunk extension (0.6% for breast surgery vs. 3.8% for thyroid surgery) but not for trunk LB between 10° and 20° (7.8% for breast surgery vs. 9.3% for thyroid surgery). The post hoc analysis revealed that most differences involved surgical assistants. In particular, as regards the percentage of time spent in static trunk flexion between 20° and 60°, we observed, for the assistants, a larger percentage in breast surgery (9.6% vs. 1.0% of thyroid surgery, *p* = 0.009, Figure 3). In contrast, during thyroid surgery, assistants spent a longer percentage of time in static trunk extension compared to those assigned to breast surgery (4.8% vs. 0.1%, *p* = 0.001, Figure 3).

The statistical analysis found no significant effect either as regards the role [F(6,18) = 1.916, *p* = 0.133, Wilks’λ = 0.610, η^2^ = 0.390] or the role × surgery interaction [F(6,18) = 1.681, *p* = 0.183, Wilks’λ = 0.641, η^2^ = 0.359], both considering trunk FE and LB.

### 3.2. Head and Neck

**Figure 4:**
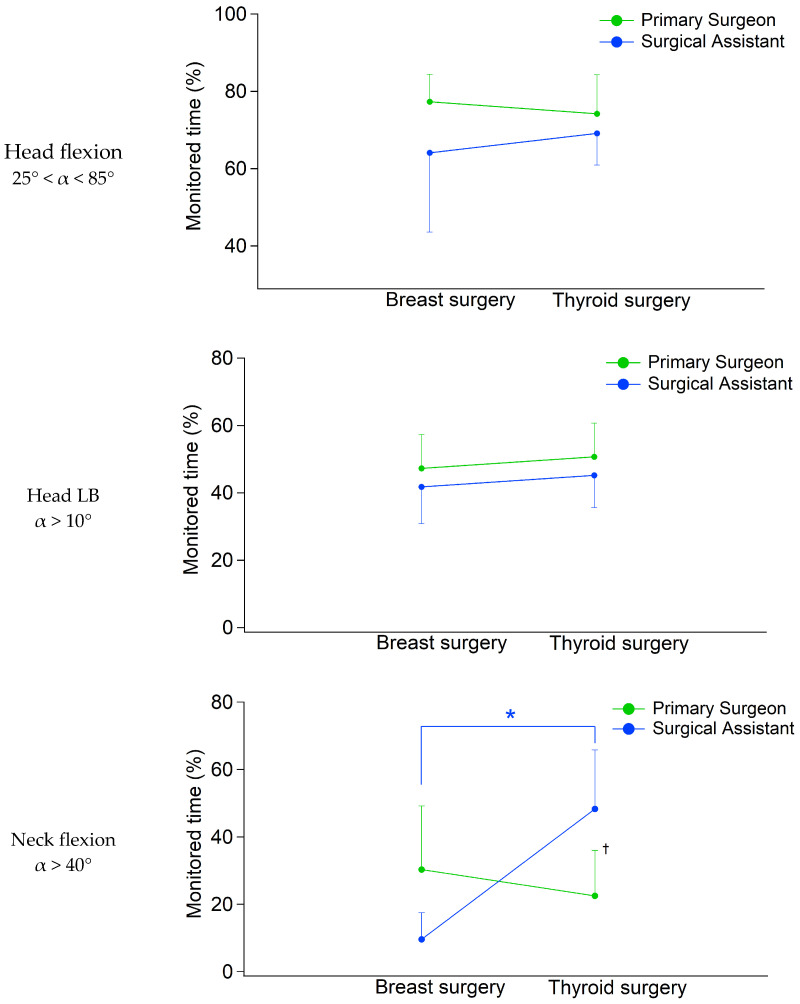
Average head flexion and LB among different surgeries and roles, following the classes of risk proposed by ISO 11226. Errors bars indicate SDs. The symbols * and † indicate, respectively, a significant difference between surgery groups within the same role and a significant main effect in the role × surgery interaction.

Regardless of the surgery type and role, surgeons typically assumed a flexed head and neck posture with mean flexion angles, respectively, of 16° and 14° and a mean LB of 9°. Figure 4 shows the percentage of the monitored time spent in head and neck non-neutral posture, according to the classes proposed by ISO 11226. The statistical analysis found a significant main effect of the surgery type [F(6,18) = 3.958, *p* = 0.011, Wilks’λ = 0.431, η^2^ = 0.569] but not of the role [F(6,18) = 1.873, *p* = 0.141, Wilks’λ = 0.616, η^2^ = 0.384]. The post hoc analysis revealed that most differences between the surgeons’ role involved the percentage of time spent in static neck flexion >40° (Figure 4). On the other hand, regardless of the type of surgery and role, surgeons spent a comparable percentage of time with static (i.e., longer than 4 s) head flexion in the range 25–85° (71.2% for breast surgery vs. 71.6% for thyroid surgery, Figure 4) and head LB above 10° (44.7% for breast surgery vs. 47.9% for thyroid surgery, Figure 4). It is noticeable that participants rarely reached extreme not-recommended head flexions, i.e., above 85° (only 2/14 breast surgeons for a maximum duration of 36 s and 10/14 thyroid surgeons up to 25 min duration). In addition, the statistical analysis found a significant main effect in the role × surgery interaction [F(6,18) = 4.072, *p* = 0.009, Wilks’λ = 0.424, η^2^ = 0.576]. Indeed, when analyzing the time spent in neck flexion > 40°, we observed an opposite trend, considering different surgeries and roles (Figure 4).

## 4. Discussion

The main purpose of the present study was to objectively assess the trunk, head and neck non-neutral postures assumed by surgeons during the performance of actual surgical procedure, as non-neutral postures were previously recognized as a co-factor implied in the development of WMSDs in this category of workers. We explored the possible effects associated with specific surgical specialties, i.e., thyroid and breast surgery. Specifically, thyroid surgery was chosen because it was classified among the specialties with the highest rates of reported musculoskeletal symptoms [10]. Breast surgery, on the other hand, was included as an example of specialty that typically requires less complex access to the surgical site. In addition, we explored the existence of possible significant differences in posture according to the role (primary or assistant) in order to clarify whether this could be considered a further risk factor. Postural data, quantified through wearable IMUs, were interpreted following the International Standard ISO 11226, which regulates exposure to static non-neutral posture.

### 4.1. Effect of the Surgical Specialty on Trunk, Head and Neck Postures

Generally speaking, our results indicated that surgeons spent most of the procedure time in static trunk posture, but rarely reached extreme flexions (i.e., >60°) and LBs (i.e., >20°). Nevertheless, during breast procedures, surgeons spent a higher percentage of time in flexion between 20° and 60°, compared to thyroid procedures (5.4% vs. 1.2%). Since previous studies suggested that excessive trunk flexion is associated with an increased risk of lower back disorders when trunk flexion exceeds 30° for more than 10% of the daily shift [23], the values reported here do not seem of particular concern.

In contrast, more alarming outcomes resulted from the analysis of the head and neck in a non-neutral posture, as our data indicated that a high percentage of time was spent in head flexion (up to 72%, between 25° and 85°) and LB (up to 48%, >10°). Moreover, it should be noted that during thyroid procedures, surgeons spent a longer time in extreme neck flexion (i.e., >40°) compared to breast surgeries (35.4% vs. 20.7%). Such values represent a critical issue since previous studies estimated that when reaching neck flexion angles between 20° and 60°, forces applied on the cervical spine increase by up to six times, with respect to the neutral position [24,25]. In addition, prolonged exposure to a non-neutral posture of the cervical/lumbar spine, despite not resulting in large muscle activation signal, may lead to excessive time spent in isometric muscular contraction, which ultimately induces relevant neuromuscular fatigue [6,24]. Thus, the peculiar head and neck postures assumed during thyroid surgery would, at least partly, explain why endocrine surgeons are particularly exposed to the development of cervical WMSDs [5,10].

The performance of open surgeries was previously associated with frequent trunk and head LBs [7,24], which occasionally reach extreme values (>10°, [26]). In our case, the primary reason for adopting asymmetrical postures was to prevent obstructing the light from the overhead operating lamp. In recent times, the use of headlamps has been proposed to solve this problem, but the relevant weight often associated with these tools may further increase the cervical loading, especially during extreme neck postures [24,27]. One possible alternative option for reducing the cervical strain while keeping an optimal lighting level within the surgical field is represented by the use of operative microscopes, which have been demonstrated effective in thyroid surgery [12], even if they are still not widespread.

Although in the literature there are no directly comparable data on these types of surgeries, previous studies regarding various specialties reported a high percentage of time spent in static trunk and neck posture [27,28,29,30], with the average neck flexion angle ranging between 19° and 40° [26,30,31]. The data of the present study appear to be in good agreement with those reported by Yang et al. [28] who found that surgeons performing different procedures (including head and neck) spent a median value of 85% of the monitored time in high-risk head/neck flexion (i.e., >20°), compared to the 72% (>25°) spent in head flexion in our study. During different gynecological surgical tasks, Zhu et al. [32] quantified a percentage of time spent in trunk flexion at >20° between 4.3% and 0.2%, which is in line with our findings for breast (5.4%) and thyroid (1.2%) surgeries. In contrast, the literature reported mixed findings for the time spent in static trunk non-neutral postures (>20°), with values reaching up to 35% [30,33].

### 4.2. Effect of the Surgical Role on Trunk, Head and Neck Posture

When considering the surgeon’s role assumed during the procedures, we observed that the higher trunk flexion measured during breast surgery was actually related to assistants’ posture (breast 9% vs. thyroid 1%). This phenomenon can be (at least partly) explained thanks to the visual monitoring performed by the professional observer during the surgeries. Indeed, it was noticed that, even though during both procedures the surgeons stood on both sides of the surgical table, in the case of breast surgery, the assistants worked in an opposite position with respect to the surgical site to facilitate access to the neoplastic area, thus bending the trunk more than the primary surgeons. In contrast, while operating on the thyroid, primary surgeons and assistants are located at the same distance with respect to the site to be operated, which may explain the similar magnitude of measured trunk flexion. Concurrently, we observed that thyroid surgeons’ assistants spent a higher percentage of time in neck flexion (>40°) compared to both breast surgeons’ assistants (48% vs. 9%, respectively) and to primary thyroid surgeons (48% vs. 22%, respectively). Nevertheless, it should be noted that breast surgeons’ assistants did not spend a higher percentage of time in neck flexion compared to the breast primary surgeon (9% vs. 30%). Therefore, it is not possible to state that surgeons’ assistants perform more demanding tasks in terms of posture; rather, it depends on the surgical specialties.

In support of our findings, the few studies that investigated the effect of the surgical role on the biomechanical risk reported different patterns of posture depending on specialty. Yurteri-Kaplan et al. [15] observed that, during gynecological surgeries, surgical assistants spent more time in trunk flexion and LB, as well as in neck flexion compared to primary surgeons, while Kant et al. [28], analyzing general and otorhinolaryngology surgery, reported a higher amount of the forward bending of the head within the primary surgeons’ group compared to assistants (i.e., instrumentation nurses). In this regard, it should be noted that the environmental conditions (especially regarding the surgical area and employed tools) are different depending on the type of the performed procedure and, as such, are potentially able to affect their posture. For instance, when performing otolaryngologist procedures, primary surgeons frequently use loupes, which require increased neck flexion [24]. Also, during gynecological procedures, surgeons stand in front of the surgical area (with the primary surgeon in a central position), thus requiring the assistants to laterally bend to reach the operating field [15]. Finally, the height of the surgical table also plays an important role in this context, since it is typically adjusted on primary surgeons’ anthropometry [34]. Despite this, it should be noted that, in the case of the present study, the only relevant change in environmental conditions was represented by the use of a step stool, which was provided to the assistants to compensate for increased table height, as previously suggested [35]. Therefore, the observed postural variations may be related to the actual role (and position) assumed by the surgeon during the procedure, with a magnitude of this effect variable according to the performed specialty. Nevertheless, the small sample size and lack of evaluation of other surgical types prevent a full generalization of the findings.

### 4.3. Strengths and Limitations

The results of this study provide new insights on ergonomic risk in surgeons and can drive a preventive approach addressed to reduce biomechanical load and to adapt the system (work environment and procedures) to the workers in such high-skilled professions. The positive consequences of such a preventive approach could impact on the fitness for work of surgeons, maintaining their health status alongside their ability to work and professionalism. The need for preventive interventions will become more urgent in the aging working population of surgeons, as reported in previous studies, where physical performance could be impaired with increasing age [36].

Saying that, some limitations of the study must be acknowledged. First, in this study, only two types of procedures were monitored. To generalize the results reported here, the same approach should be applied to other surgical specialties (possibly including a larger sample size) which could confirm these findings or introduce new risk factors. Second, even if it is known that females are at higher risk of developing WMSDs [6], the small size of the surgical team in this study limited our ability to analyze gender-based differences among surgeons. In addition, to reduce variability, it would be beneficial to monitor the same surgeons across different surgeries and roles, an approach we were unable to implement due to the small sample size.

## 5. Conclusions

In conclusion, the quantitative approach based on a limited number of IMUs adopted in this study appeared to be well tolerated by surgeons. Thus, it might represent a valid tool to support the assessment of ergonomic risks associated with various surgical procedures and roles by providing data that are easily interpretable in accordance with current international standards (ISO 11226). The data obtained confirm that, by spending most of the procedure time in a standing posture with extreme forward neck flexion, surgeons are exposed to a high risk of developing WMSDs, especially when frequently performing thyroid surgeries. Moreover, our results suggest that the role assumed by the surgeon might influence the risk of developing WMSDs at the neck and trunk, even though the effect of this factor is likely to be dependent on the type of the performed surgery. The use of operative microscopes might partially mitigate the risk associated with the surgical procedure, allowing the introduction of sitting periods while reducing the cervical strain. Nevertheless, the cost associated with the implementation of this new tool makes this approach not yet widespread when performing endocrine procedures. Rotating roles during procedures within the work shifts (from primary to assistant and vice versa) could also help to reduce the biomechanical risk by evenly distributing the more hazardous workload among surgeons. However, further investigations are needed to understand the effect of implementing these strategies on overall postural load. Future studies should ideally perform such analyses on a larger scale, incorporating additional surgical specialties and considering individual factors such as gender, age and work seniority.

## Figures and Tables

**Table 1:** Participant demographic and anthropometric characteristics. Values are expressed as mean (SD).

	Thyroid Surgery		Breast Surgery	
	Primary Surgeons	Surgical Assistants	Primary Surgeons	Surgical Assistants
Participants	7M	7M	7M	7 (6M, 1F)
Age (years)	55.7 (3.4)	40.4 (9.0) †	50.8 (9.2)	56.1 (6.4) *
Height (cm)	175.7 (0.4)	172.4 (4.1)	173.4 (2.3)	167.6 (4.6) *,†
Body mass (kg)	78.4 (7.8)	71.9 (10.9)	79.0 (10.4)	64.3 (14.2) †
Seniority at work (years)	30.9 (5.4)	10.4 (7.9) †	18.7 (13.1) *	18.7 (9.3)

F: female; M: male. The symbol * denotes a statistically significant difference vs. the group “thyroid surgery” within the same role while the symbol † denotes a statistically significant difference vs. the group “primary surgeons” within the same type of surgery. Both differences were determined using an unpaired *t*-test (*p* < 0.05).

**Table 2:** Classes of risk proposed in the standard ISO 11226 to evaluate the static (e.g., longer than 4 s) non-neutral posture of the trunk, head and neck.

Body Segment	Movement	ISO 11226 Class	Description
Trunk	Extension	α < 0°	Not recommended
	Flexion	0° < α < 20°	Always acceptable
		20° < α < 60°	Conditionally acceptable
		α > 60°	Not recommended
	Lateral Bending (+ → right;− → left)	0° < α < 10°	Always acceptable
		10° < α < 20°	Not recommended
		α > 20°	Not recommended
Head	Extension	α < 0°	Not recommended
	Flexion	0° < α < 25°	Always acceptable
		25° < α < 85°	Conditionally acceptable
		α > 85°	Not recommended
	Lateral Bending ^a^ (+ → right;− → left)	0° < α < 10°	Always acceptable
		α > 10°	Not recommended
Neck	Extension	α < 0°	Not recommended
	Flexion	α < 40°	Always acceptable
		α > 40°	Not recommended

^a^ ISO 11226 does not distinguish between head and neck lateral bending.

## Data Availability

The data presented in this study are available on request from the corresponding author.

## Supplementary Materials

The following supporting information can be downloaded at https://www.mdpi.com/article/10.3390/bioengineering12030299/s1: Figure S1: Average trunk FE and LB, head FE and LB and neck flexion among different surgeries and roles, following the classes of risk proposed by ISO 11226. Errors bars indicate SDs and the symbol * indicates a significant difference between surgery groups within the same role; Table S1: Descriptive results of trunk, head and neck non-neutral postures following the classes of risk proposed in the standard ISO 11226 for different surgical specialties and roles. Values are reported as means (SD).

## Abbreviations

The following abbreviations are used in this manuscript:

WMSDs	Work-related Musculoskeletal Disorders;
MIS	Minimally Invasive Surgeries;
IMUs	Inertial Measurement Units;
FE	Flexion–Extension;
LB	Lateral Bending
