# Supplementary material for: Use of Wearable Inertial Sensors to Assess Trunk and Cervical Postures Among Surgeons: Effect of Surgical Specialties and Roles
Source: Bioengineering (Basel). 2025 Mar 15;12(3):299. doi: 10.3390/bioengineering12030299 (PMC11939344; doi:10.3390/bioengineering12030299)
Supplement: Supplementary file 1 [file bioengineering-12-00299-s001.zip › bioengineering-3496954-supplementary.pdf]

Supplementary Material

Table S1. Descriptive results of trunk, head and neck non-neutral postures following the classes of risk proposed in the standard ISO 11226 for different surgical specialties and roles. Values are reported as means (SD).

**Body segment**	**Movement**	**ISO 11226 class**	**% of time spent in non-neutral posture**				**% of time spent in static non-neutral posture**			
			**Breast surgery**		**Thyroid surgery**		**Breast surgery**		**Thyroid surgery**	
			Primary	Assistant	Primary	Assistant	Primary	Assistant	Primary	Assistant
Trunk	Extension	$\alpha < 0°$	2.5 (2.4)	1.0 (0.5)	5.1 (3.9)	6.1 (4.1)	0.9 (1.0)	0.2 (0.3)	2.9 (2.6)	4.8 (3.7)
	Flexion	$20° < \alpha < 60°$	2.2 (3.0)	10.8 (7.9)	2.0 (2.6)	1.6 (2.6)	1.9 (2.9)	9.5 (7.1)	1.5 (2.5)	1.1 (2.1)
		$\alpha > 60°$	0.0	0.0	0.0	0.0	0.0	0.0	0.0	0.0
	Lateral Bending	$10° < \alpha < 20°$	10.5 (9.4)	6.1 (5.0)	15.1 (10.2)	4.9 (4.3)	9.9 (9.1)	5.5 (4.7)	14.2 (9.9)	4.4 (4.4)
		$\alpha > 20°$	1.1 (1.6)	1.2 (1.1)	0.9 (1.3)	0.8 (1.0)	1.0 (1.6)	1.1 (1.1)	0.9 (1.3)	0.8 (1.0)
Head	Extension	$\alpha < 0°$	1.0 (1.0)	3.9 (6)	3.2 (2.8)	1.1 (0.7)	0.2 (0.4)	1.9 (3.7)	1.1 (1.2)	0.3 (0.5)
	Flexion	$25° < \alpha < 85°$	77.5 (7.1)	64.4 (20.4)	74.4 (10.1)	69.3 (8.3)	77.3 (7.1)	64.1 (20.5)	74.2 (10.1)	69.1 (8.2)
		$\alpha > 85°$	0.0	0.0	0.0	0.1 (0.2)	0.0	0.0	0.0	0. (0.2)
	Lateral Bending	$\alpha > 10°$	58.0 (8.4)	51.2 (9.7)	60.1 (7.4)	52.2 (9.3)	47.3 (10.0)	41.8 (10.9)	50.8 (10.1)	45.2 (9.6)
Neck	Extension	$\alpha < 0°$	1.5 (1.2)	4.4 (6.4)	4.2 (3.3)	1.4 (0.9)	0.3 (0.4)	1.9 (3.7)	1.4 (1.4)	0.4 (0.4)
	Flexion	$\alpha > 40°$	30.3 (18.9)	9.7 (7.9)	22.6 (13.5)	48.5 (17.6)	30.3 (18.9)	9.7 (7.9)	22.5 (13.5)	48.4 (17.5)

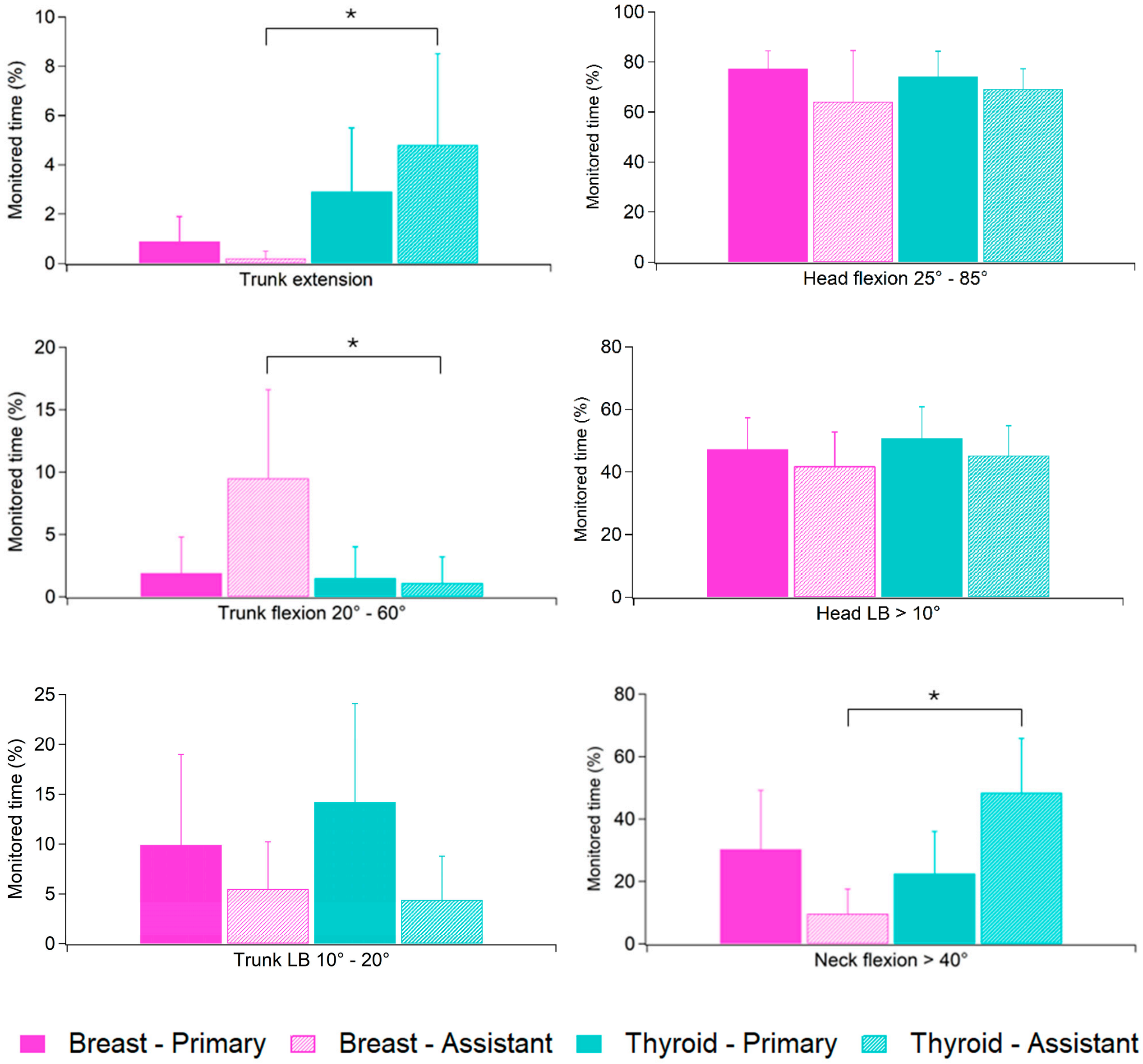

Figure S1. Average trunk FE and LB, head FE and LB and neck flexion among different surgeries and roles, following the classes of risk proposed by ISO 11226. Errors bars indicate SDs and the symbol * indicates a significant difference between surgery groups within the same role.
